# Artificial Intelligence Assisted Smartphone System for Real-Time Detection and Severity Assessment of Digital Clubbing

**DOI:** 10.7150/ijms.126125

**Published:** 2026-03-23

**Authors:** Wei-Hsun Wang, Guan-Tsen Liu, Su-Juan Chen, Yu-Chu Chiang, Hsiao-Hsuan Wang, Yong-Hong Chiang, Yu-Tzu Hu, Wen-Shin Hsu

**Affiliations:** 1 Department of Post-Baccalaureate Medicine, College of Medicine, National Chung Hsing University, Taichung 402202, Taiwan.; 2 Department of Golden-Ager Industry Management, Chaoyang University of Technology, Taichung 413310, Taiwan.; 3 Department of Orthopedic Surgery, Changhua Christian Hospital, Changhua 500209, Taiwan.; 4 Department of Medical Imaging and Radiology, Shu-Zen Junior College of Medicine and Management, Kaohsiung 821, Taiwan.; 5 Department of Medical Information, Chung Shan Medical University, Taichung 402201, Taiwan.; 6 Informatics Office Technology, Chung Shan Medical University Hospital, Taichung 402201, Taiwan.; 7 Program for Precision Health and Intelligent Medicine, Graduate School of Advanced Technology, National Taiwan University, Taipei 106319, Taiwan.

**Keywords:** digital clubbing, artificial intelligence, deep learning, smartphone screening, cardiopulmonary disease.

## Abstract

Digital clubbing is an important clinical sign associated with a range of cardiopulmonary diseases; however, its detection and severity assessment in routine practice largely rely on subjective visual inspection. This study proposes an automated, smartphone-based system for real-time detection and severity assessment of digital clubbing using deep learning techniques. The system integrates the YOLOv8 object detection model for initial clubbing classification, the KeypointNet model for anatomical landmark localization, and a novel Clubbing Fingers Severity Analysis (CFSA) algorithm to quantify the Lovibond angle and grade disease severity.

Finger images were acquired using a smartphone camera with an OpenCV-based preprocessing strategy to standardize finger-to-camera distance and improve image consistency. Model performance was evaluated using publicly available anonymized datasets. The proposed system achieved an overall accuracy of 94.7% for digital clubbing detection and severity classification. The YOLOv8 model attained a classification accuracy of 92.5%, while the KeypointNet model achieved a landmark localization accuracy of 96.5%. Notably, the recall for severe digital clubbing reached 94.0%, indicating strong sensitivity for identifying high-risk cases.

By providing real-time, non-invasive, and reproducible assessments, the proposed system addresses the limitations of conventional visual examination and supports objective severity grading. Although further clinical validation is required, this smartphone-based approach demonstrates strong potential as a preliminary screening support tool for early identification of digital clubbing in clinical and community-based settings, particularly in resource-limited environments.

## Introduction

Lung diseases and cardiovascular diseases remain leading causes of morbidity and mortality worldwide, contributing substantially to the burden of chronic illness 1. Increasing evidence indicates that localized or systemic inflammatory responses in chronic lung diseases may be closely linked to the development of cardiovascular conditions 2. Cardiovascular diseases (CVD), primarily ischemic heart disease (IHD) and stroke, represent major causes of global mortality and disability 3. Similarly, chronic obstructive pulmonary disease (COPD) is a significant public health issue, with underdiagnosis and misclassification leading to underestimation of its true severity 4. In the United States, COPD ranks as the fourth leading cause of death 5 and imposes heavy economic and healthcare burdens 6-7. Both COPD and CVD are often asymptomatic in early stages, delaying detection and treatment and resulting in progression to more severe complications 8-9.

Digital clubbing, characterized by bulbous enlargement of the fingertips, is one of the few visible signs that may indicate early cardiopulmonary disease 10. It is strongly associated with multiple conditions including lung cancer, COPD, cyanotic congenital heart disease, and idiopathic pulmonary fibrosis, with underlying mechanisms linked to vascular endothelial growth factor and platelet-derived growth factor 11. Its diagnostic value has long been emphasized in both pulmonary and cardiac contexts 12, and its presence can also reflect the course of underlying disease processes 13. Despite its clinical importance, recognition of digital clubbing remains challenging. Visual inspection alone is subjective and prone to error 14, contributing to missed or delayed diagnoses.

Traditional clinical tests for clubbing include the Schamroth window test 15 and the Lovibond angle measurement 16,17. While widely used, both methods rely heavily on naked-eye assessment and can yield inconsistent results, especially in subtle or early cases. These limitations underscore the need for objective, reproducible, and accessible methods to assess digital clubbing.

Recent advances in artificial intelligence (AI) and deep learning have enabled automated image-based analysis in diverse medical applications, ranging from lung nodule detection to skin lesion classification 18,31. Convolutional neural networks (CNNs) and related architectures have achieved high accuracy in disease recognition tasks 19-24. In addition, comprehensive reviews in computer vision have highlighted the rapid evolution of deep learning architectures for object detection and image classification 25. Among these approaches, the YOLOv8 model has demonstrated strong generalization and high-speed detection performance in real-time image analysis tasks, including biomedical and clinical imaging applications 26-29,38,39, while KeypointNet provides accurate localization of anatomical landmarks 30. Building upon these advances, this study proposes an AI-assisted smartphone system that integrates YOLOv8 for detecting digital clubbing and KeypointNet for localizing anatomical landmarks required for Lovibond angle calculation. A custom Clubbing Finger Severity Analysis (CFSA) algorithm is further applied to classify severity into clinically relevant categories based on established diagnostic criteria 16,17.

The objective of this study is to develop and validate a real-time, non-invasive tool for the detection and severity assessment of digital clubbing. By enabling reliable recognition from smartphone-acquired images, this system has the potential to support clinicians in early identification of cardiopulmonary diseases and to provide accessible community-level screening, thereby reducing the risk of delayed diagnosis and disease progression.

## Materials and Methods

**Figure 1:**
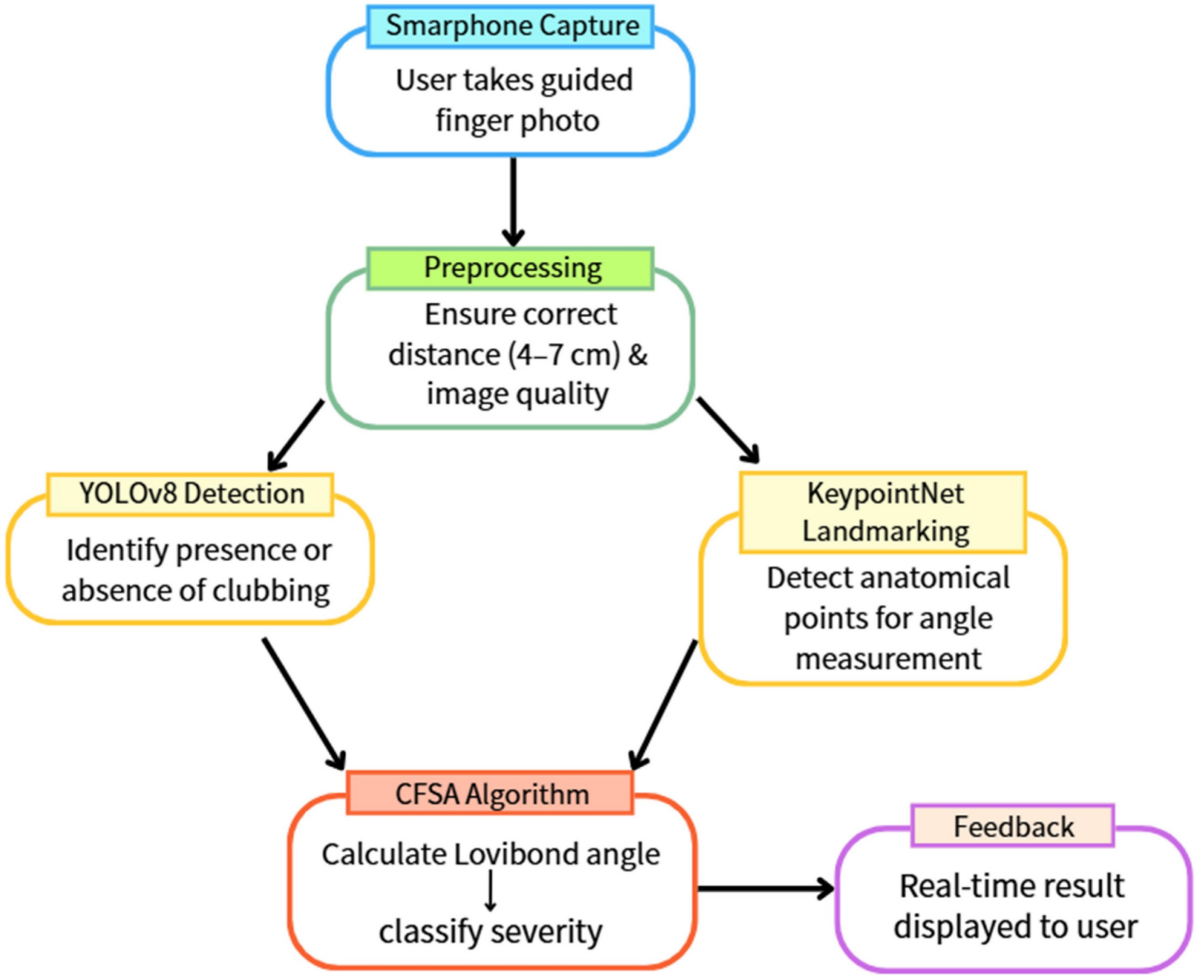
Overall architecture of the proposed artificial intelligence-assisted system for detecting and grading digital clubbing.

Figure 1 illustrates the overall architecture of the proposed system, encompassing both the model training and real-time inference workflows. All deep learning models were trained and evaluated in a personal computer environment prior to deployment for mobile-based screening applications.

For image acquisition, users are guided by a smartphone application that provides on-screen posture instructions to ensure consistent finger positioning. To further standardize image quality, an OpenCV-based algorithm is employed to estimate the distance between the finger and the camera in real time. Image capture is automatically triggered only when the finger is positioned within a predefined optimal range, thereby reducing variability related to scale and focus.

Once captured, the images are processed sequentially by two integrated deep learning models. The YOLOv8 model first performs binary classification to determine the presence or absence of digital clubbing. Images identified as positive are subsequently analyzed by the KeypointNet model, which localizes the anatomical landmarks required for severity assessment. The Clubbing Fingers Severity Analysis (CFSA) algorithm then computes the Lovibond angle based on these landmarks and categorizes disease severity into clinically relevant levels. The final assessment results are displayed immediately within the mobile application, enabling real-time, non-invasive preliminary screening.

### Image Acquisition

In this study, digital clubbing was categorized into four clinically relevant severity levels: normal, mild, moderate, and severe. The classification criteria were defined according to established Lovibond angle thresholds reported in prior clinical literature 16,17.

Model development and evaluation were conducted using publicly available and anonymized finger images obtained from open-access online medical image repositories. All images were used in accordance with their respective licensing terms and usage policies. No additional clinical data collection or direct interaction with human subjects was required.

Inclusion criteria required clear lateral or oblique views of the distal phalanx with a visible nail contour and sufficient resolution to allow accurate landmark localization. Images with severe occlusion, excessive blur, incomplete nail structures, or indistinct anatomical boundaries were excluded from analysis.

The final dataset comprised 1,956 images of clubbed fingers and 1,744 images of normal fingers. Severity labeling was performed based on Lovibond angle thresholds defined in established diagnostic standards 16,17. No demographic metadata (e.g., age, sex, ethnicity, or geographic origin) were available in the open-access dataset. Consequently, demographic stratification analysis could not be performed. This limitation may influence generalizability across diverse populations and is further discussed in the Discussion section.

### Image Preprocessing

Accurate detection of digital clubbing relies on consistent image quality during acquisition. Variations in the distance between the finger and the camera may affect image scale, focus, and anatomical feature visibility, thereby influencing model performance. To improve consistency, an OpenCV-based preprocessing module was implemented to guide standardized image capture.

During acquisition, the system estimates the relative distance between the finger and the camera by analyzing the proportion of the finger region within the image frame. Image capture is automatically triggered only when the detected finger size falls within a predefined optimal range. If the finger is positioned too close or too far from the camera, real-time on-screen prompts instruct the user to adjust the distance accordingly.

To determine the optimal acquisition range, validation experiments were conducted across distances ranging from 1 cm to 10 cm under controlled lighting conditions. Although recognition performance gradually decreased at extreme distances due to scale variation and focus limitations, distances between 4 cm and 7 cm consistently yielded stable and high recognition accuracy for both classification and keypoint localization tasks. Minor variability across trials was observed, reflecting natural fluctuations in lighting and device positioning. Based on these findings, the application was configured to restrict image capture to the 4-7 cm range to improve input consistency and enhance the robustness of subsequent detection and severity assessment.

### Model Based on YOLOv8

To determine whether an input image exhibited digital clubbing, the YOLOv8 deep learning model was employed. YOLOv8 is a state-of-the-art one-stage object detection framework known for its high accuracy and low inference latency, making it suitable for real-time clinical screening applications 25-29,39. Compared with earlier YOLO variants, YOLOv8 incorporates architectural refinements that enhance feature extraction efficiency while maintaining strong discriminative performance.

In this study, YOLOv8 was trained using 3,700 annotated finger images with a binary classification objective (clubbing vs. normal). Model training was performed using the AdamW optimizer with a batch size of 20 over 150 epochs. Model performance was evaluated on an independent test dataset using standard classification metrics, including accuracy, precision, recall, and F1-score, derived from confusion matrix analysis.

**Figure 2:**
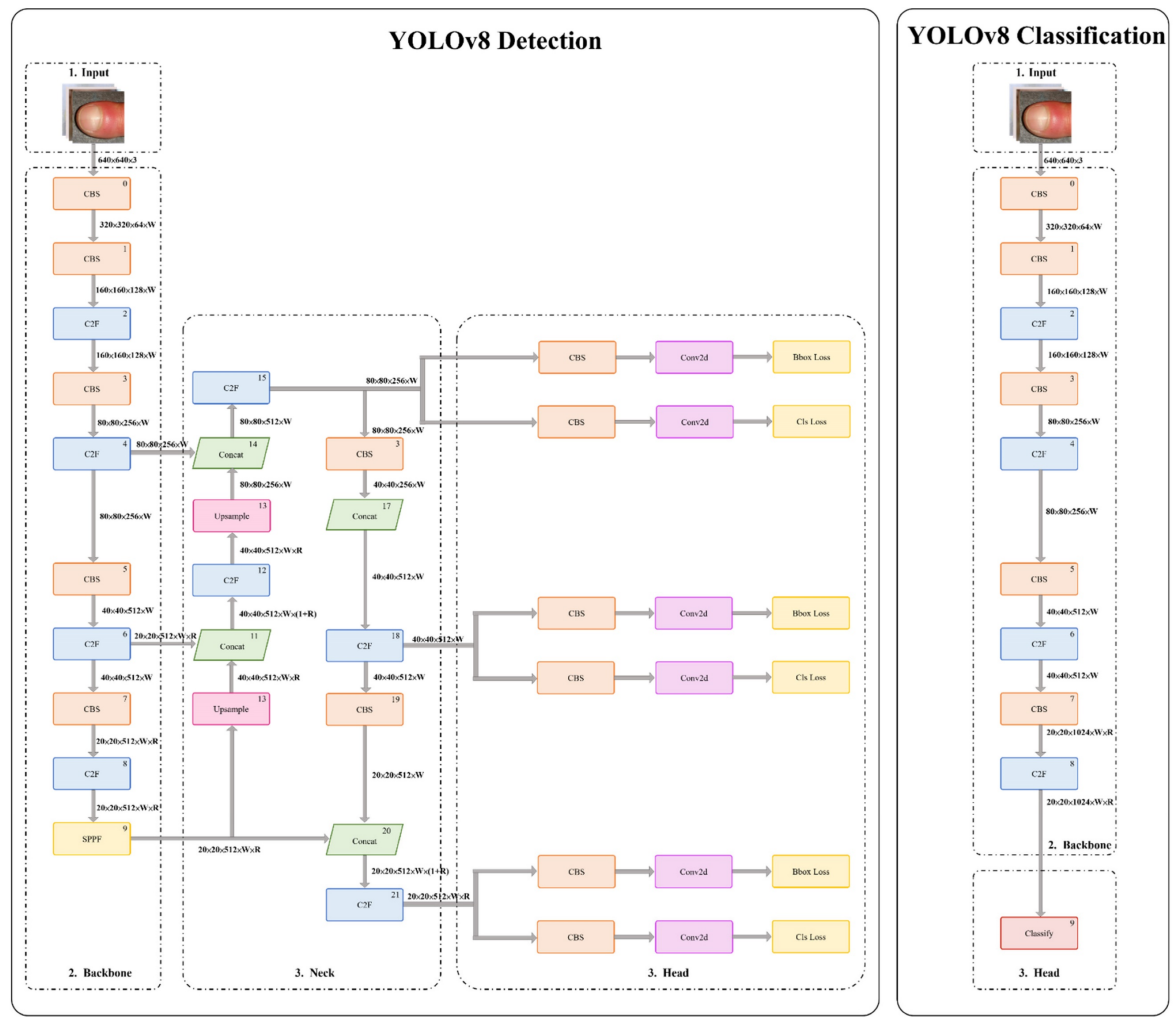
Architecture of the YOLOv8-based model.

As illustrated in Figure 2, the YOLOv8 architecture comprises a backbone for hierarchical feature extraction and a detection head for classification and localization. An anchor-free detection strategy combined with non-maximum suppression was applied to generate final predictions with high accuracy and computational efficiency. Detailed layer-wise architecture specifications and training hyperparameters are provided in Appendix B.

Model optimization was performed using a multi-component loss function designed to balance localization accuracy and classification reliability. Detailed mathematical formulations and parameter definitions are provided in Appendix A.

### Model Based on KeypointNet

In this study, three anatomical landmarks were consistently defined as the nail matrix, proximal nail fold, and nail plate for Lovibond angle computation. To enable automated severity assessment of digital clubbing, the KeypointNet deep learning model was employed to localize these landmarks and provide the geometric information necessary for objective severity grading. KeypointNet has demonstrated strong performance in keypoint-based learning tasks and is well suited for extracting clinically relevant structural features from medical images.

**Figure 3:**
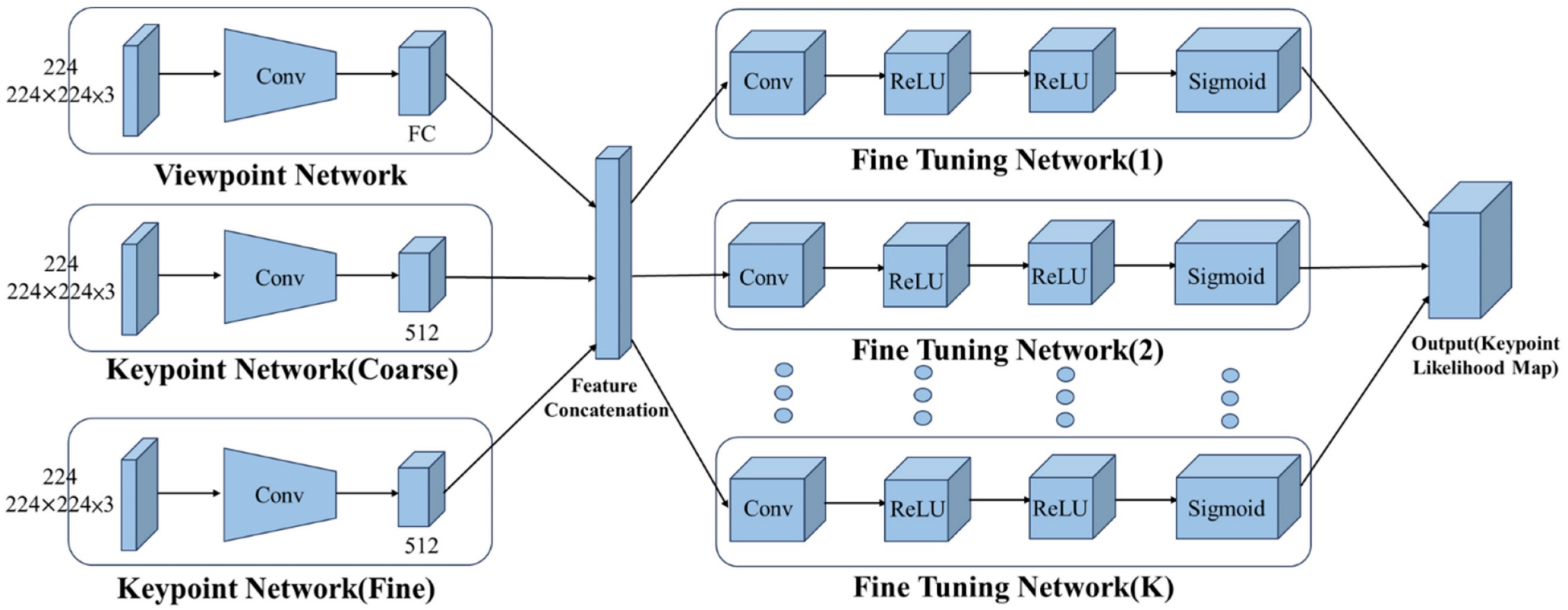
Architecture of the KeypointNet-based model.

As illustrated in Figure 3, the KeypointNet architecture comprises a multi-branch feature encoder and a feature decoder. The encoder, built on a convolutional neural network backbone, extracts semantically meaningful image representations. A coarse-to-fine prediction strategy is applied to progressively refine keypoint positions, while dedicated subnetworks adjust each landmark location to improve localization precision. The final landmark positions are determined based on probability maps generated by the network.

The model was configured with optimized training parameters to achieve robust and precise landmark localization. Detailed architectural specifications and hyperparameters are provided in Appendix C. Model optimization employed a combined loss function incorporating point-wise localization accuracy and geometric consistency to preserve the angular relationships among the three anatomical landmarks. Detailed mathematical formulations are provided in Appendix A.

This optimization strategy enables precise and stable keypoint localization, which is essential for reliable Lovibond angle-based severity grading in clinical screening.

### Clubbing Fingers Severity Analys Algorithm, CFSA

To provide an objective and reproducible assessment of digital clubbing severity, this study developed the Clubbing Fingers Severity Analysis (CFSA) algorithm, which automates the traditional Lovibond angle inspection. The Lovibond method, originally described by Lovibond 16, relies on visual estimation of the angle formed between the nail plate and the proximal nail fold of the distal phalanx. Although clinically valuable, visual assessment is inherently subjective and prone to inter-observer variability.

**Figure 4:**
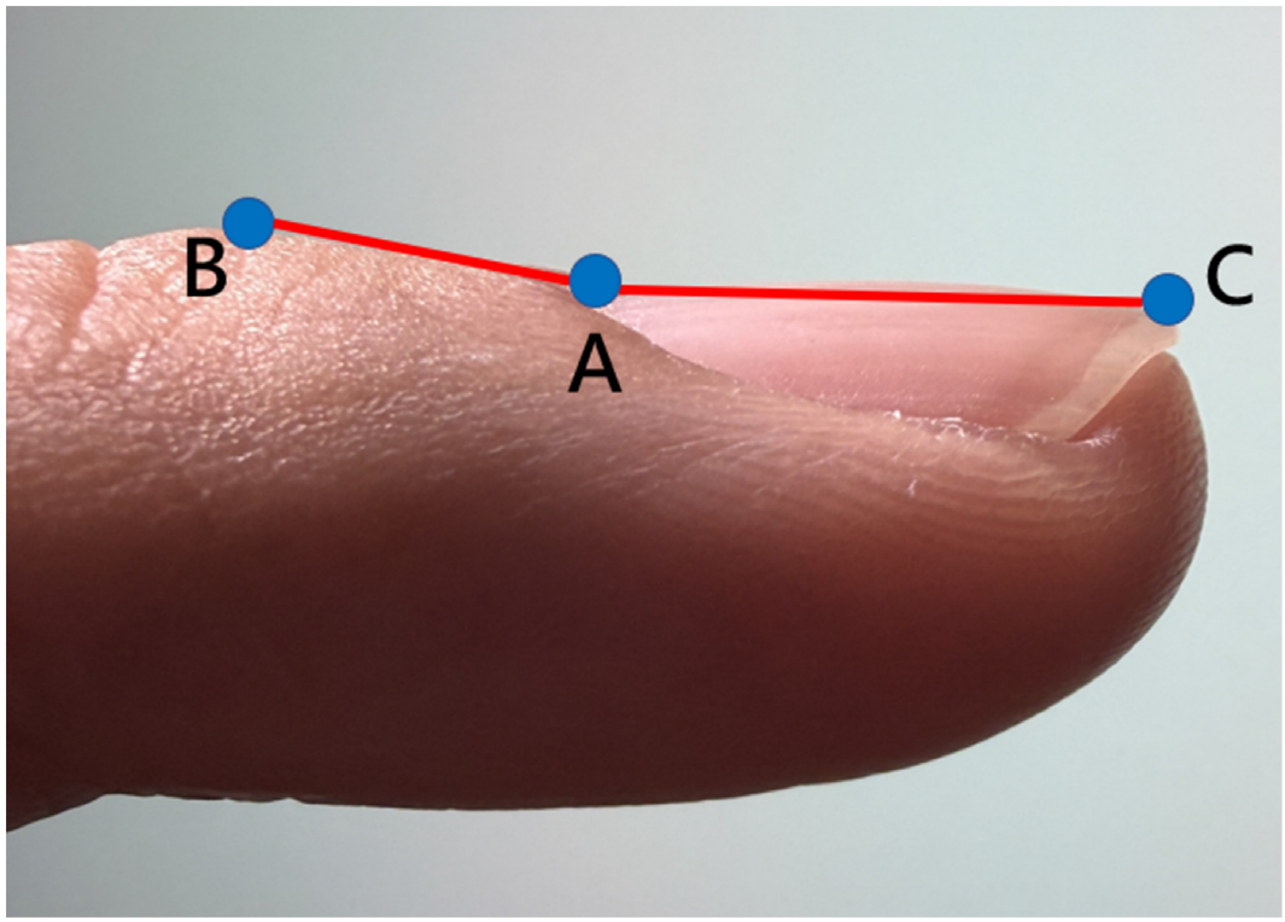
The three anatomical landmarks used for Lovibond angle measurement. Point A: Nail matrix; Point B: Proximal nail fold; Point C: Nail plate.

**Figure 5:**
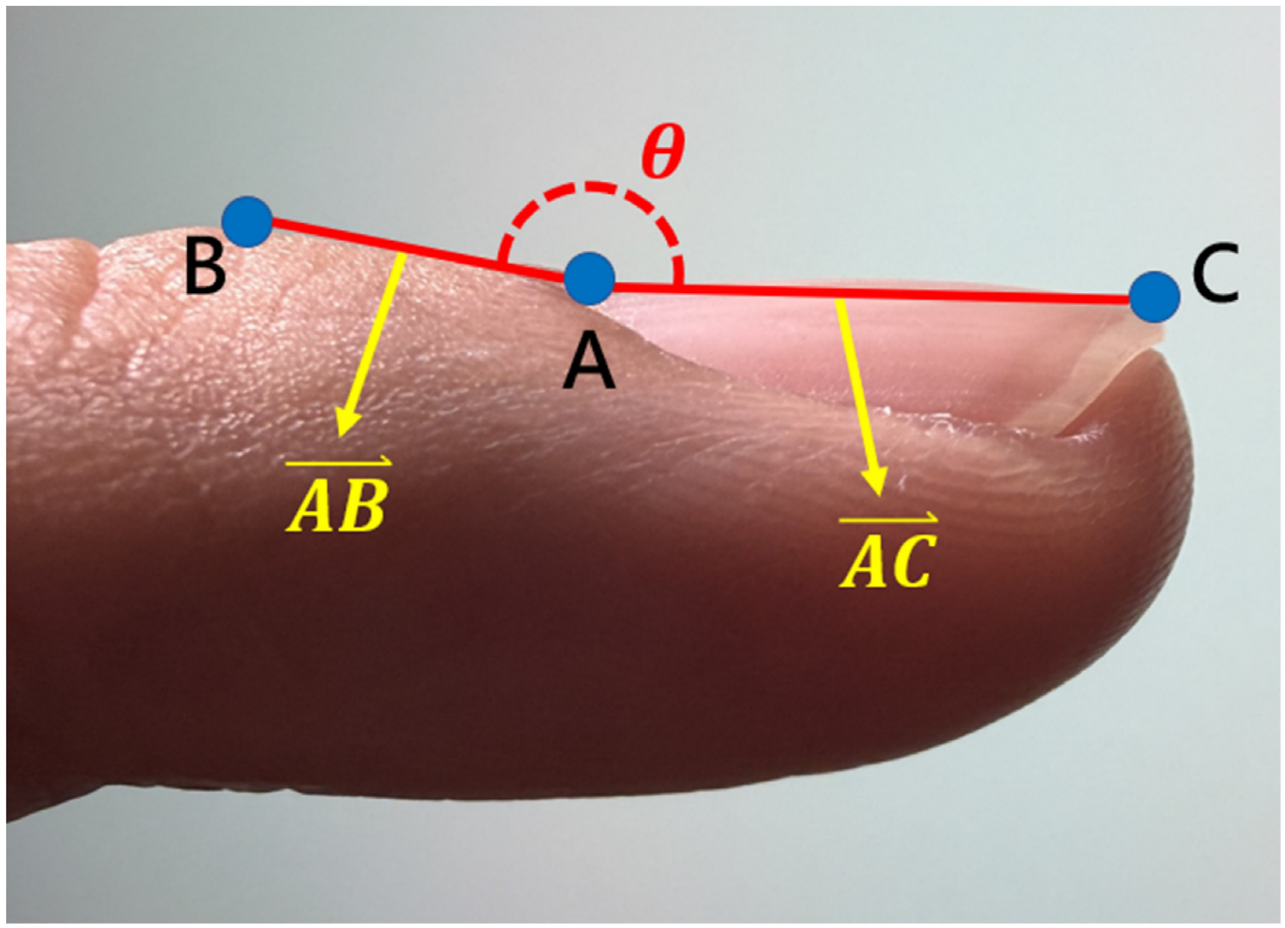
Geometric representation of the Lovibond angle calculation based on three anatomical landmarks. Point A (nail matrix), Point B (proximal nail fold), and Point C (nail plate) form the triangle used for angle computation.

The CFSA algorithm addresses these limitations by leveraging the three anatomical landmarks localized by the KeypointNet model: the nail matrix, proximal nail fold, and nail plate. Using these landmarks, a geometric triangle is constructed for each input image, and the Lovibond angle is calculated automatically based on the geometric relationships among the three points, as illustrated in Figures 4 and 5.

Based on established clinical criteria 16,17,40, digital clubbing severity is categorized into four levels according to predefined angle thresholds, as summarized in Table 5. An angle of 160° corresponds to normal morphology, angles between 160° and 180° indicate mild clubbing, an angle of 180° represents moderate clubbing, and angles exceeding 180° are classified as severe clubbing. Increasing angle values correspond to more advanced morphological changes.

For images classified as normal by the YOLOv8 model, the system directly assigns a “normal” label and bypasses severity grading. This design reduces unnecessary computation and ensures that severity analysis is applied only when digital clubbing is detected.

By integrating deep learning-based anatomical landmark localization with a rule-based algorithm grounded in established clinical standards, the CFSA algorithm enables objective, reproducible, and real-time quantification of digital clubbing severity. Detailed mathematical formulations are provided in Appendix A.

### Performance Evaluation

The performance of the proposed system was evaluated using confusion matrix analysis on an independent test dataset. Correctly identified digital clubbing cases were classified as true positives (TP), while missed detections were recorded as false negatives (FN). Correctly identified normal cases were counted as true negatives (TN), and normal cases incorrectly classified as clubbing were recorded as false positives (FP). TP, TN, FP, and FN denote true positives, true negatives, false positives, and false negatives, respectively.

To comprehensively assess classification performance, standard diagnostic metrics were calculated, including accuracy, precision, recall, and F1-score. These evaluation metrics are widely adopted in medical artificial intelligence research because they reflect clinically meaningful trade-offs between false-positive and false-negative predictions.

In screening applications, particular emphasis is placed on recall to minimize missed detection of individuals with potential underlying cardiopulmonary disease. At the same time, precision and F1-score provide complementary information regarding classification reliability and overall balance between sensitivity and specificity.

Detailed mathematical definitions of the evaluation metrics are provided in Appendix A4.

## Results

The proposed system integrates two deep learning models: YOLOv8 for binary classification and KeypointNet for anatomical landmark localization, followed by a rule-based severity grading algorithm. Performance was evaluated separately for each component and for the integrated severity classification system.

All experiments were conducted using a workstation equipped with an Intel i5-12400 8-core CPU, 16 GB RAM, and an NVIDIA RTX 2050 GPU, implemented in PyTorch. Although model training was performed on this workstation, the deployed system is designed to operate on standard consumer smartphones, ensuring practical applicability in real-world clinical and community settings.

**Figure 6:**
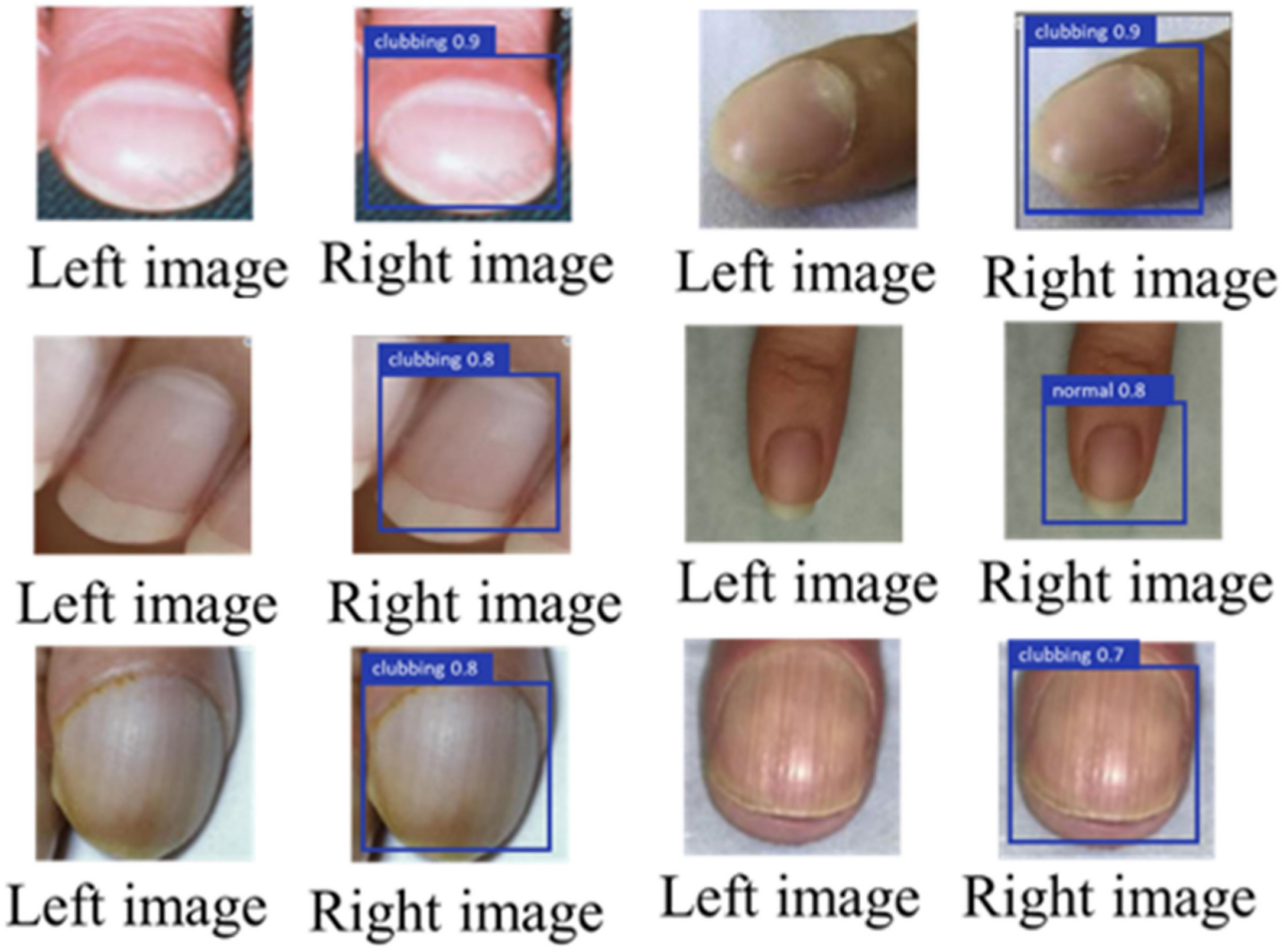
Labeled image before being input into YOLOv8(left), and the result after YOLOv8 detection(right).

**Figure 7:**
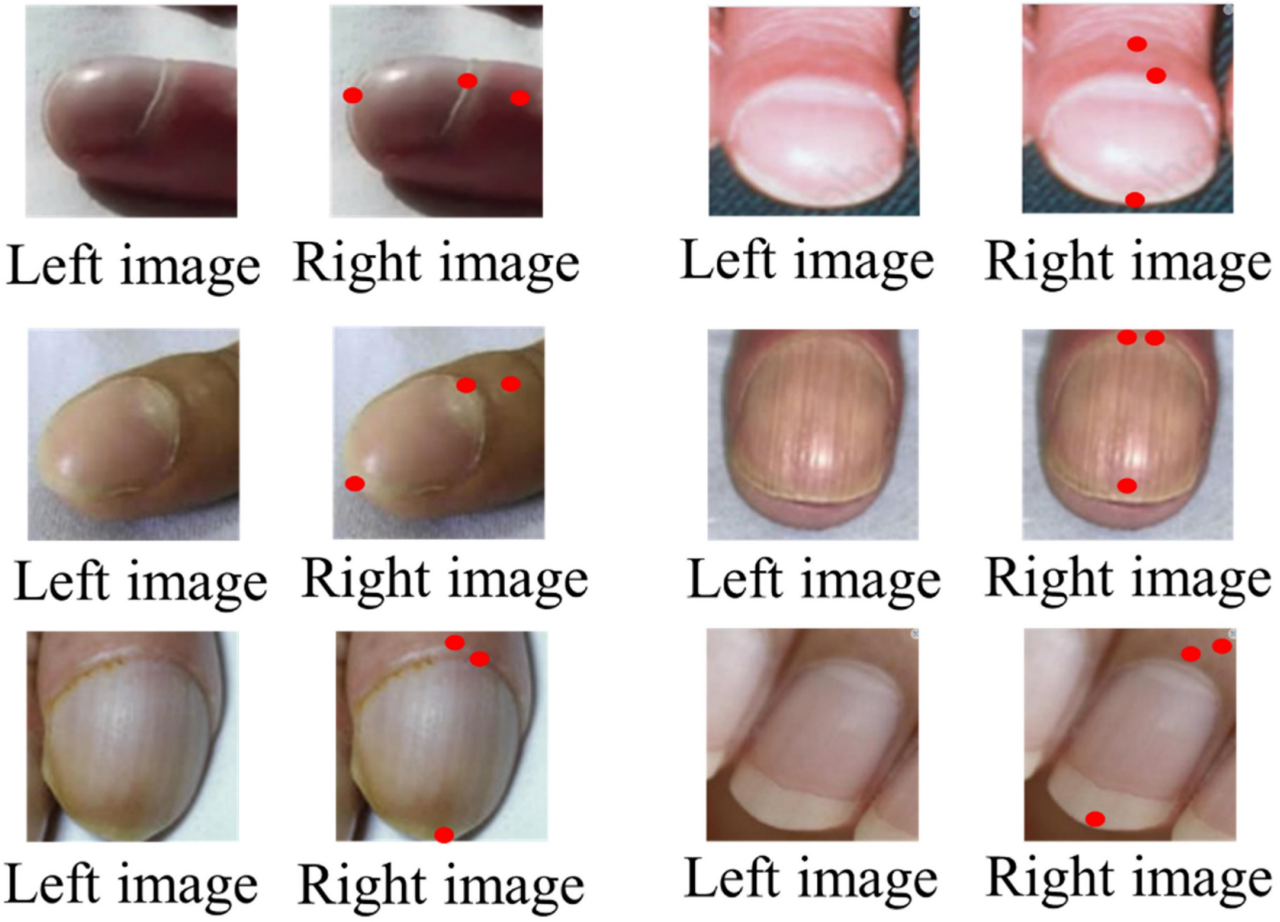
Labeled image before being input into KeypointNet(left), and the result after KeypointNet detection(right).

Figures 6 and 7 illustrate representative outputs of the integrated system. YOLOv8 successfully distinguished between normal and clubbed fingers, while KeypointNet accurately localized the three predefined anatomical landmarks: the nail matrix, proximal nail fold, and nail plate. These outputs confirm that the models generate both technically accurate and clinically interpretable results required for Lovibond angle-based severity grading.

### YOLOv8 Binary Classification Performance

The YOLOv8 model was evaluated using 3,700 images, including 1,956 images of clubbed fingers and 1,744 images of normal fingers. Table 6 presents the confusion matrix for binary classification. Performance metrics derived from the confusion matrix are summarized in Table 7.

The high recall indicates reliable detection of true clubbing cases, while the strong precision reflects a low false-positive rate. The balanced F1-score demonstrates stable binary classification performance suitable for screening applications.

### KeypointNet Landmark Localization Performance

The KeypointNet model was evaluated on 1,956 images of clubbed fingers for localization of three anatomical landmarks: nail matrix, proximal nail fold, and nail plate. Performance metrics are summarized in Table 9.

The results demonstrate stable and accurate localization across all three anatomical landmarks, with the highest performance observed for nail plate detection.

### Effect of Data Augmentation on Landmark Localization

To evaluate robustness under variable imaging conditions, data augmentation techniques including random flipping, brightness adjustment (±20%), and scaling (±10%) were applied only to the training set. The validation and test sets were kept unchanged to ensure a fair comparison. Table 10 summarizes the confusion matrix of the KeypointNet model trained with augmentation and evaluated on the same test set. Performance metrics are reported in Table 11. Following augmentation-based training, the model showed a modest improvement in overall precision and F1-score, suggesting enhanced robustness for landmark localization under variable imaging conditions.

Following augmentation, performance improved modestly, indicating enhanced robustness of landmark localization under variable imaging conditions.

### Integrated Severity Classification Performance

The integrated system combines YOLOv8 detection, KeypointNet landmark localization, and the CFSA algorithm for four-level severity classification (normal, mild, moderate, severe). Performance metrics are summarized in Table 13.

The integrated system demonstrates balanced and reliable performance across all severity levels, with particularly strong performance in normal and severe categories. The slight decrease in overall accuracy compared with binary classification reflects the increased complexity of multi-level severity discrimination.

## Discussion

In this study, YOLOv8 was first applied for binary classification to determine the presence or absence of digital clubbing, followed by KeypointNet-based landmark annotation for severity assessment. To verify whether the system provides advantages over existing methods, separate analyses were conducted for each model, and their performance was compared against other commonly used architectures under the same dataset. Additional evaluations included testing the effect of finger placement distance and comparing the system with existing diagnostic approaches to determine its overall strengths and limitations. Since the system is designed to provide immediate feedback, computational efficiency and real-time performance were also assessed.

Table 14 presents the performance comparison of YOLOv8 with other models. The YOLOv8 model achieved a classification accuracy of 95.5%, a precision of 95.9%, and a mean Average Precision(mAP) of 93.8%, with the highest overall accuracy among the tested models. While the ensemble Mask R-CNN model achieved slightly higher mAP, its computational latency and large model size significantly limit its suitability for real-time applications. Similarly, EfficientNet-B0 demonstrated robust precision but is less optimal for rapid deployment in time-sensitive environments.

From a clinical perspective, the balanced performance of YOLOv8 across speed, accuracy, and reliability makes it particularly suited for real-time screening of digital clubbing. This is critical in practical scenarios where rapid and accurate assessment may support early recognition of underlying cardiopulmonary disease. By contrast, models with higher latency or excessive resource demands may be less feasible for integration into smartphone-based applications or community screening tools.

To further evaluate the robustness of the chosen architecture, the performance of YOLOv8 was compared with other versions within the YOLO series. Finger images, both normal and with digital clubbing, were processed using YOLOv7, YOLOv8, and YOLOv9. Table 15 presents the comparative results.

The YOLOv8 model achieved a classification accuracy of 95.5%, a precision of 95.9%, and a mean Average Precision (mAP) of 93.8%. Although YOLOv9 demonstrated slightly higher benchmark accuracy in controlled testing (Table 15), YOLOv8 provided a more balanced combination of classification performance, computational efficiency, and deployment readiness for real-time clinical applications. YOLOv7, while reliable, exhibited comparatively lower accuracy and recall.

From a clinical perspective, consistency and stability are of greater importance than marginal improvements in benchmark scores. YOLOv8 provided a more balanced combination of accuracy, efficiency, and robustness, making it more suitable for real-time clinical screening of digital clubbing. This reliability is critical when integrating AI models into smartphone-based diagnostic tools, where stable performance across diverse conditions directly impacts clinical trust and usability. Inference times in Table 15 were measured under batch processing conditions for YOLO-series comparison experiments, whereas Table 14 reports single-image inference time under real-time deployment settings.

Based on the performance comparison results, YOLOv8 demonstrated superior classification accuracy, precision, and recall compared with other tested models, confirming its suitability for the initial detection of digital clubbing.

In addition, KeypointNet was applied to annotate the three anatomical landmarks required for Lovibond angle measurement, thereby enabling automated severity assessment. To evaluate its relative performance, images of clubbed fingers were processed with several alternative models, and their results were compared under the same conditions. The outcomes are summarized in Table 16.

KeypointNet achieved a landmark annotation accuracy of 96.5%, a mean Average Precision(mAP) of 97.2%, and an F1-score of 0.96. Across all evaluated metrics including accuracy, precision, average precision, F1-score, and recall KeypointNet consistently outperformed other models.

From a clinical perspective, the high accuracy and stability of KeypointNet are particularly significant, as precise localization of anatomical landmarks directly determines the reliability of Lovibond angle calculation. This ensures that severity grading is both objective and reproducible, reducing the variability inherent in traditional visual inspection. Consequently, KeypointNet provides a robust foundation for real-time clinical screening and monitoring of digital clubbing severity.

Accurate recognition of digital clubbing is a critical task, and since the system is designed to provide immediate feedback after image capture, the processing speed of the deep learning models is a major consideration. Table 17 summarizes the efficiency-related metrics of the YOLOv8 model. The inference time was approximately 10-12 ms on a GPU and 50-70 ms on a CPU, with computational complexity of 28-30 GFLOPs.

Although YOLOv8 requires higher computational complexity compared with YOLOv5 and is similar to YOLOv7, architectural improvements such as the C2f module and lightweight head design resulted in a 20%-30% increase in inference speed. By contrast, EfficientDet-B0, while competitive in terms of accuracy, demonstrated substantially slower inference times, limiting its feasibility for real-time use.

From a clinical perspective, these findings are significant. Rapid inference enables the system to deliver results within milliseconds, ensuring that users whether patients in a community setting or clinicians in a busy outpatient clinic receive immediate and reliable feedback. This responsiveness is essential for screening applications, where delayed results could reduce usability and hinder clinical decision-making. Collectively, the results confirm that YOLOv8 combines both accuracy and efficiency, making it particularly well suited for real-time implementation in clinical and community health environments.

In addition to image classification, this study employed the KeypointNet model to perform landmark annotation as a preliminary step for severity assessment. Once YOLOv8 identified an image as showing digital clubbing, it was immediately processed by KeypointNet to localize the three anatomical points required for Lovibond angle measurement. Since severity grading depends directly on accurate and timely keypoint localization, both processing time and computational complexity of KeypointNet were evaluated.

Table 18 summarizes the time-efficiency metrics of the model. KeypointNet achieved an inference time of 8 ms on GPU and 35 ms on CPU, with a computational complexity of only 2 GFLOPs. Compared with alternative approaches, BlazePose offered slightly faster processing but at the cost of reduced accuracy, while OpenPose provided competitive accuracy but required substantially greater computational resources, limiting feasibility on hardware-constrained devices.

From a clinical perspective, the performance of KeypointNet is noteworthy. Its combination of high accuracy and low latency ensures that Lovibond angle measurement can be obtained in real time, even on standard consumer devices. This responsiveness is essential for practical deployment in both clinical and community settings, where immediate feedback supports early recognition of digital clubbing and facilitates timely medical evaluation.

Through the comprehensive comparison of time efficiency between YOLOv8 and KeypointNet, it can be concluded that integrating both models into a single system provides accurate image classification and anatomical landmark annotation with minimal processing delay, making it the optimal choice for real-time screening applications.

To further ensure consistency and reliability during image capture, the system incorporates OpenCV-based preprocessing. Using the smartphone camera, the algorithm estimates the distance between the finger and the lens based on the relative size of the finger within the frame, thereby standardizing input quality.

Table 19 summarizes the performance of YOLOv8 at various capture distances. The highest accuracy (mean Average Precision, mAP > 98.5%) was achieved when the finger was placed between 1-3 cm from the lens; however, this range occasionally introduced occlusion or lighting artifacts. A distance of 4-7 cm maintained similarly high accuracy (mAP 97.5%-98.3%) while minimizing interference, with inference times of 40-47 ms, which remain clinically acceptable. Beyond 8 cm, accuracy gradually decreased (mAP 96.5%-97.2%) and the error rate increased (up to 3.5%).

From a clinical perspective, these findings suggest that positioning the finger between 4-7 cm from the smartphone camera provides the optimal balance between accuracy and usability. This practical guideline ensures that the system can deliver reliable results in both clinical settings and community-based self-assessments, even under variable lighting and environmental conditions.

After confirming that YOLOv8 achieves optimal classification accuracy when finger images are captured at a distance of 1-7 cm, it was necessary to further evaluate the effect of capture distance on the KeypointNet model, since both models contribute directly to the overall detection outcome. Table 20 presents the classification accuracy of KeypointNet at various finger-to-camera distances.

When the finger was positioned 1-3 cm from the lens, accuracy was exceptionally high (mAP > 98.8%, error rate < 1%). However, this very close range occasionally introduced problems such as image blur and uneven lighting, which could compromise the precision of landmark annotation. At 4-7 cm, accuracy remained consistently high (mAP 98.7%-98.2%) with negligible error, and landmark localization was more stable under variable conditions. At 8-10 cm, accuracy began to decline (mAP 97.5%-98.0%) and error rates increased (up to 3.0%).

From a clinical perspective, these findings highlight that maintaining a 4-7 cm capture distance provides the optimal balance between precision and robustness for keypoint annotation. This guideline ensures that Lovibond angle measurements remain accurate and reproducible, even when images are captured under non-ideal conditions, such as in home or community environments.

It should be noted that the mAP values reported in Tables 19 and 20 reflect controlled distance-specific evaluations rather than overall classification accuracy across heterogeneous datasets.

Based on the combined evaluation of YOLOv8 and KeypointNet, optimal and stable accuracy was achieved when finger placement was maintained between 4-7 cm from the camera. To ensure consistent image quality, the system was therefore designed to use OpenCV to detect finger position during capture and to restrict image acquisition to this optimal range. This design choice ensures uniform input conditions, which are critical for maintaining reliable and reproducible diagnostic performance.

It is noteworthy that performance trends across different capture distances exhibited relatively smooth transitions. This behavior can be attributed to the controlled acquisition protocol implemented in the proposed system. Specifically, OpenCV-based distance estimation, standardized image cropping, and preprocessing normalization were applied consistently across all experimental settings, thereby minimizing random environmental variability. Furthermore, the evaluation dataset was collected under structured experimental conditions rather than uncontrolled real-world scenarios. As a result, performance degradation with increasing distance followed a gradual and predictable pattern instead of abrupt fluctuations. Future validation under more heterogeneous clinical and community environments will further assess system robustness.

Beyond binary detection and landmark localization, the clinical relevance of the system ultimately depends on reliable severity stratification. Severity classification was performed using the CFSA algorithm, which is based on Lovibond angle thresholds. The performance results are summarized in Table 21. The algorithm achieved an overall accuracy of 94.7%, confirming its ability to reliably differentiate among severity levels.

From a clinical perspective, several findings are noteworthy. First, the model demonstrated high precision in identifying asymptomatic and mild cases, supporting early detection and timely medical referral. Second, for the severe category, the recall rate reached 94.0%, indicating high sensitivity in detecting high-risk patients who require urgent evaluation. Finally, although the moderate category is inherently challenging to distinguish from mild or severe cases, the model still achieved precision and recall values above 91%, demonstrating robustness and balanced performance across all levels.

Collectively, these results highlight the clinical utility of the CFSA algorithm: it not only minimizes the risk of missed severe cases but also provides consistent grading across all severity levels, thereby supporting risk stratification, disease monitoring, and timely intervention in both clinical and community settings.

In this study, a smartphone-based system was developed to enable convenient and accessible detection of digital clubbing. Finger images captured by the user are analyzed in real time using the YOLOv8 model for binary classification, KeypointNet for anatomical landmark localization, and the CFSA algorithm for severity grading, with results displayed directly on the mobile interface. This approach was designed to provide a simple, user-friendly solution for early identification of digital clubbing in both clinical and community settings.

Upon reviewing current detection methods related to clubbing-associated diseases, it was noted that no existing online tools or smartphone-based systems are specifically designed for digital clubbing detection. Traditional methods, such as low-dose computed tomography (LDCT) 43 and the Schamroth window test 44, present important limitations. LDCT, while sensitive, is costly, resource-intensive, and not suitable for routine or community-based screening. The Schamroth test, although simple, is highly subjective and prone to inter-observer variability. More general AI-based tools, such as Google DermAssist 45, do not specifically target clubbing and lack the precision required for Lovibond angle-based severity assessment.

To systematically evaluate the advantages of the proposed system, a functional comparison was conducted against these existing approaches using the ISO/IEC 25010:2023 framework 46, focusing on five quality characteristics: functional suitability, timeliness, usability, security, and portability. The results, summarized in Table 22, demonstrate that the proposed system outperforms existing diagnostic methods in all evaluated aspects.

By integrating deep learning models into a portable smartphone-based platform, the proposed system achieves high diagnostic accuracy while offering immediate, non-invasive, and reproducible results. This represents a clinically meaningful improvement over traditional methods, with the potential to support early recognition, risk stratification, and timely intervention for cardiopulmonary diseases associated with digital clubbing.

The performance evaluation of the proposed system demonstrated that integrating YOLOv8 for binary detection, KeypointNet for landmark localization, and CFSA for severity grading results in high accuracy and stability across all tasks. With an overall severity classification accuracy of 94.7% and a recall of 94.0% for severe cases, the system effectively minimizes the risk of missed diagnoses in patients with advanced disease while maintaining balanced performance across mild and moderate cases. These findings suggest that the system can provide reliable, objective grading that surpasses the subjectivity of traditional visual inspection.

From a clinical perspective, the ability to provide immediate, reproducible, and non-invasive results is particularly valuable in settings where advanced diagnostic tools are unavailable. In community or primary care environments, the system can serve as a screening support tool, enabling early recognition of potential cardiopulmonary disease and guiding timely referrals. For patients with chronic conditions such as COPD or congenital heart disease, the system may also be applied in longitudinal monitoring, allowing physicians to track changes in digital clubbing severity over time.

Compared with existing approaches, the proposed system offers several advantages. Unlike LDCT, it requires no specialized equipment or exposure to radiation. Unlike the Schamroth test, it reduces reliance on subjective interpretation. Unlike general-purpose dermatology AI tools, it is tailored to digital clubbing detection and incorporates severity grading based on clinically validated criteria. Its smartphone-based design ensures portability and accessibility, making it feasible for use in both high-resource hospitals and resource-limited settings.

Despite the encouraging performance demonstrated by the proposed system, several limitations should be acknowledged. First, the models were trained and evaluated primarily using publicly available, anonymized open-access datasets. Although such datasets are valuable for algorithm development and benchmarking, they may not fully capture the diversity encountered in real-world clinical settings. In particular, the absence of detailed demographic metadata (e.g., age, sex, ethnicity) limits the ability to assess performance variability across different patient populations. Potential sources of bias, including differences in skin tone, finger morphology, nail characteristics, and age-related anatomical variation, as well as variations in image quality, lighting conditions, and acquisition devices, may influence the generalizability of the proposed approach. Future work will involve prospective data collection in collaboration with healthcare institutions to construct a clinically annotated dataset with documented demographic information. Such validation studies will enable more rigorous evaluation of system robustness across diverse populations and clinical environments.

From a clinical workflow perspective, the proposed smartphone-based system is intended to serve as a preliminary screening support tool rather than a definitive diagnostic device. It may be deployed in outpatient clinics, primary care settings, or community health programs to facilitate rapid identification of individuals with suspected digital clubbing. Individuals flagged by the system can subsequently be referred for further clinical evaluation and confirmatory diagnostic procedures, such as imaging or specialist consultation, thereby supporting early detection and more efficient allocation of healthcare resources.

Accordingly, system performance may vary when applied in heterogeneous clinical or community environments. Future work will focus on expanding the training dataset to include more diverse populations and imaging conditions, as well as conducting prospective clinical validation studies to further assess robustness, generalizability, and real-world clinical applicability.

While the proposed system demonstrates strong performance in structured experimental settings, the present study should be regarded as a methodological feasibility evaluation rather than a definitive clinical validation trial. The dataset was derived from publicly available anonymized image repositories without confirmed clinical diagnoses. Therefore, prospective validation using clinically annotated cases will be required to further establish real-world diagnostic reliability.

## Conclusions

This study developed an integrated smartphone-based system that combines the YOLOv8 object detection model, the KeypointNet keypoint localization model, and a custom Clubbing Fingers Severity Analysis (CFSA) algorithm to enable rapid and objective detection of digital clubbing with automated severity grading. The proposed system achieved an overall severity classification accuracy of 94.7%, with YOLOv8 attaining a binary classification accuracy of 95.5% and KeypointNet achieving a landmark localization accuracy of 96.5%. Importantly, the recall for severe cases reached 94.0%, suggesting the system's potential to reduce the likelihood of missed high-risk cases during preliminary screening.

By providing real-time, non-invasive, and reproducible assessment results, the proposed system offers advantages over conventional approaches such as the Schamroth window test and subjective visual inspection. Its portability and computational efficiency make it suitable for deployment in outpatient clinics, primary care settings, and community-based screening programs, particularly in resource-limited environments where access to specialized diagnostic tools may be limited.

Nevertheless, several limitations should be acknowledged. The system was primarily trained and evaluated using publicly available datasets, which may not fully represent the diversity encountered in real-world clinical practice. Variability in skin tone, hand morphology, nail characteristics, environmental lighting conditions, and image acquisition devices may influence detection performance. Although stable results were observed under controlled experimental settings, further validation in heterogeneous clinical environments is necessary.

Future work will focus on expanding dataset diversity, incorporating prospective clinical validation studies, and evaluating integration within real-world clinical workflows. Additional physiological or multimodal features may also be explored to enhance diagnostic robustness and clinical applicability.

In summary, the proposed system represents a promising auxiliary screening tool for the early detection and monitoring of digital clubbing. By enabling objective and standardized severity assessment, it may support earlier identification of underlying cardiopulmonary conditions and contribute to improved clinical management strategies.

## Figures and Tables

**Table 5:** Classification criteria for digital clubbing severity based on Lovibond angle assessment

Grading	Standard
Normal	 = 160°
Mild	160° 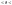 180°
Moderate	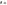 180°
Severe	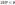

**Table 6:** Real-time confusion matrix for YOLOv8-based digital clubbing classification

Actual Class \ Predicted Class	Clubbing Fingers	Normal
Clubbing Fingers	1867	89
Normal	79	1665

**Table 7:** Performance metrics of the YOLOv8 model for digital clubbing classification

Metric	Value
Accuracy	95.46%
Precision	95.94%
Recall	95.45%
F1-Score	95.69%

**Table 8:** Confusion matrix for KeypointNet-based anatomical landmark localization

Actual Class \ Predicted Class	Nail Plate	Nail matrix	Proximal nail fold
Nail Plate	1888	39	29
Nail matrix	61	1794	101
Proximal nail fold	42	46	1868

**Table 9:** Evaluation metrics for KeypointNet landmark localization

Metrics \ Category	Nail Plate	Nail matrix	Proximal nail fold	Average
Accuracy	96.52%	91.72%	95.50%	94.58%
Precision	94.83%	95.47%	93.49%	94.59%
Recall	96.52%	91.72%	95.50%	94.58%
F1-Score	95.67%	93.55%	94.48%	94.57%

**Table 10:** Confusion matrix after data augmentation

Actual Class \ Predicted Class	Nail Plate	Nail matrix	Proximal nail fold
Nail Plate	1893	35	28
Nail matrix	50	1815	91
Proximal nail fold	38	40	1878

**Table 11:** Performance metrics after data augmentation

Metrics \ Category	Nail Plate	Nail matrix	Proximal nail fold	Average
Accuracy	96.78%	92.79%	96.01%	95.19%
Precision	95.56%	96.03%	94.04%	95.21%
Recall	96.78%	92.79%	96.01%	95.19%
F1-Score	96.16%	94.38%	95.02%	95.19%

**Table 12:** Confusion matrix for digital clubbing severity classification using the integrated system

Actual Class\Predicted Class	Normal	Mild	Moderate	Severe
Normal	1694	40	10	0
Mild	10	598	42	2
Moderate	8	34	588	22
Severe	0	8	20	624

**Table 13:** Performance metrics for multi-level digital clubbing severity classification

Category	Precision	Recall	F1-Score
Normal	98.9%	97.0%	97.9%
Mild	88.0%	91.6%	89.8%
Moderate	90.7%	86.3%	88.5%
Severe	96.2%	95.7%	96.0%
Overall Accuracy: 94.7%			
Macro-Average F1-Score: 93.0%			

**Table 14:** Performance comparison of YOLOv8 with other deep learning models for digital clubbing classification

Model	Accuracy	Precision	mAP	Inference Time	Model Size	F1-Score	Recall
**YOLOv8**	**95.5%**	**95.9%**	**93.8%**	**12ms**	**25MB**	**95.7%**	**95.5%**
EfficientNet-B0	94.2%	93.1%	92.4%	30ms	29MB	91.0%	91.2%
ResNet	90.7%	89.6%	87.8%	20ms	35MB	88.0%	91.2%
Single Mask R-CNN	90.0%	89.0%	91.0%	400ms	40MB	92.0%	88.0%
Ensemble Mask R-CNN	95.0%	94.0%	96.0%	500ms	45MB	91.0%	93.0%

**Table 15:** Performance comparison of YOLOv8 with other YOLO series models for digital clubbing classification

Model	Accuracy	Precision	mAP	Inference Time	Model Size	F1-Score	Recall
YOLOv9	96.5%	95.8%	95.2%	22ms	27MB	95.0%	94.5%
**YOLOv8**	**95.5%**	**95.9%**	**93.8%**	**18ms**	**25MB**	**95.7%**	**95.5%**
YOLOv7	95.2%	92.8%	91.7%	15ms	30MB	91.5%	92.0%
YOLOv6	92.3%	91.0%	88.5%	12ms	35MB	89.0%	90.5%
YOLOv5	91.5%	90.2%	86.9%	13ms	45MB	88.0%	89.0%

**Table 16:** Performance comparison of KeypointNet with other keypoint detection models

Model	Accuracy	Precision	mAP	Inference Time	Model Size	F1-Score	Recall
**KeypointNet**	**96.5%**	**95.8%**	**97.2%**	**30ms**	**50MB**	**96.0%**	**94.9%**
ResNet	89.7%	88.3%	87.4%	22ms	35MB	86.0%	90.0%
YOLOv8	93.2%	93.7%	89.3%	14ms	25MB	90.0%	92.4%
Faster R-CNN	90.0%	92.5%	89.2%	28ms	40MB	88.0%	91.5%
EfficientNet	92.1%	90.5%	89.0%	18ms	20MB	89.0%	91.0%

**Table 17:** Computational efficiency and inference time comparison of the YOLOv8 model

Model	Inference Time (GPU)	Inference Time (CPU)	FLOPs	Model Size	FPS (GPU)
**YOLOv8**	**10~12ms**	**50~70ms**	**28~30GFLOPs**	**22MB**	**80~90**
YOLOv5	12~15ms	60~80ms	17GFLOPs	14MB	65~75
YOLOv7	13~17ms	70~90ms	25GFLOPs	20MB	60~70
EfficientDet-B0	35~40ms	150~180ms	39GFLOPs	15MB	25~28
MobileNetV3+SSD	20~25ms	90~120ms	10~15GFLOPs	12MB	40~50

**Table 18:** Computational efficiency and inference time comparison of the KeypointNet model

Model	Inference Time (GPU)	Inference Time (CPU)	FLOPs	Model Size	FPS (GPU)
**KeypointNet**	**8ms**	**35ms**	**2GFLOPs**	**12MB**	**125**
BlazePose	10~15ms	60~80ms	~3GFLOPs	3.5MB	60~70
PoseNet	20~30ms	100~130ms	~5GFLOPs	9MB	30~40
AlphaPose	25~35ms	120~160ms	~25GFLOPs	50MB	30~35
OpenPose	40~60ms	180~250ms	40~50GFLOPs	90MB	15~20

**Table 19:** Effect of finger-to-camera distance on YOLOv8 classification performance

Distance	mAP	F1-Score	Error Rate
1 cm	98.9%	0.99	0.6%
2 cm	98.7%	0.98	0.9%
3 cm	98.5%	0.98	1.1%
4 cm	98.3%	0.97	1.4%
5 cm	98.1%	0.97	1.6%
6 cm	97.8%	0.96	2.0%
7 cm	97.5%	0.96	2.3%
8 cm	97.2%	0.95	2.7%
9 cm	96.8%	0.94	3.2%
10 cm	96.5%	0.94	3.5%

**Table 20:** Effect of finger-to-camera distance on KeypointNet keypoint localization performance

Distance	mAP	F1-Score	Error Rate
1 cm	99.2%	0.99	0.5%
2 cm	99.0%	0.98	0.8%
3 cm	98.8%	0.98	1.0%
4 cm	98.7%	0.97	1.2%
5 cm	98.6%	0.97	1.5%
6 cm	98.4%	0.96	1.8%
7 cm	98.2%	0.96	2.0%
8 cm	98.0%	0.95	2.5%
9 cm	97.7%	0.95	2.8%
10 cm	97.5%	0.94	3.0%

**Table 21:** Performance of digital clubbing severity classification using the CFSA algorithm

Grading	Accuracy	Precision	Recall
Normal	95.0%	94.2%	93.5%
Mild	94.5%	93.1%	92.0%
Moderate	93.8%	92.3%	91.2%
Severe	95.6%	94.8%	94.0%

**Table 22:** Functional comparison of the proposed smartphone-based system with existing methods for digital clubbing assessment

	Functionality	Immediacy	Usability	Safety	Portability
**Proposed Method**	**Automated detection and severity assessment of digital clubbing**	**Real-time analysis**	**User-friendly smartphone interface**	**Non-invasive**	**Mobile application**
Low-Dose CT	Detection of underlying cardiopulmonary abnormalities	Requires scheduled examination	Requires specialized medical equipment	Low-dose radiation exposure	Not portable
Shamroth's Window Test	Visual screening for advanced digital clubbing	Immediate manual assessment	Operator-dependent and subjective	Non-invasive	Portable
Google DermAssist	Image-based assessment of selected skin conditions	Dependent on image submission and processing	Requires internet access and compatible device	Non-invasive	Mobile application

**Table 1:** In the YOLOv8-based model, stage *i* has 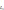
layers, with Input Resolution 〈
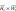
*〉* and Output Channels 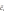.

Stage *i*	Operator 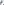	Resolution 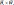	Channels 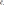	Layer 
1	Conv3×3	640×640	32	1
2	Conv3×3	320×320	64	1
3	C2f3×3	160×160	128	2
4	Conv3×3	80×80	256	1
5	C2f3×3	40×40	512	2
6	SPPF3×3	20×20	512	1
7	C2f3×3	40×40	256	2
8	Conv3×3	20×20	512	1
9	C2f3×3	20×20	1024	2

**Table 2:** Hyperparameters used for training

Hyperparameters	Selected Values
Loss function	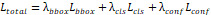
Optimizer	AdamW
Learning rate	1× 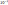
Batch size	20
Epoch	150

**Table 3:** Describes the resolution and number of channels for each layer

Stage *i*	Operator 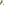	Resolution 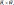	Channels 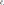	Layer 
1	Conv3×3	224×224	64	1
2	Conv3×3	112×112	128	1
3	Conv3×3	56×56	256	1
4	Conv3×3	28×28	512	1
5	Fine Output	28×28	K	-
6	Conv3×3	224×224	64	1
7	Conv3×3	112×112	128	1
8	Conv3×3	56×56	256	1
9	Fine Output	28×28	K	-
10	Fine Tune(×K)	28×28	64→1	3(per K)
11	Final Heatmap	28×28	K	-

**Table 4:** Hyperparameters used for training

Hyperparameters	Selected Values
Loss function	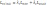
Optimizer	AdamW
Learning rate	1× 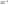
Batch size	20
Epoch	150

## Data Availability

The datasets used and analyzed during the current study were derived from publicly available and anonymized image repositories. The original data sources remain publicly accessible in accordance with their respective usage policies. Due to redistribution restrictions associated with the original repositories, the compiled dataset generated during this study is not publicly redistributed. However, detailed information regarding dataset composition, inclusion criteria, labeling procedures, and preprocessing methods is provided within the manuscript to ensure methodological transparency and reproducibility.

## Appendix A. Mathematical Formulations

### A1. YOLOv8 Loss Functions

To optimize detection performance, a composite loss function was employed.

(1) Bounding Box Loss

Where denotes the predicted bounding box, denotes the ground-truth bounding box.

(2) Classification Loss

Where is the ground-truth class label, is the predicted probability.

(3) Confidence Loss

Where represents the ground-truth objectness label and represents the predicted confidence score.

(4) Total YOLO Loss

where , , and are weighting coefficients.

### A2. KeypointNet Loss Functions

To ensure accurate landmark localization and geometric consistency, the KeypointNet model was optimized using a combined loss.

(5) Mean Absolute Error

Where *N=3* in this study (three anatomical landmarks), is the ground-truth keypoint coordinate, and is the predicted keypoint coordinate.

(6) Angle Consistency Loss

Where is the annotated angle, and , , are the coordinates of the three keypoints.

(6) Final Keypoint Loss

Where and are weighting parameters.

### A3. CFSA Geometric Formulation

Let the three anatomical landmarks be defined as:

(8)-(10) Side Lengths

(11) Cosine Law

cos(θ)=

(12) Angle Calculation

The same formulation is applied to compute .

(13) Lovibond Angle

Where max (,,) represents the largest internal angle of the triangle.

### A4. Performance Evaluation Metrics

(14) Accuracy =

(15) Precision =

(16) Recall =

(17) F1-Score = 2

## Appendix B. YOLOv8 Architecture Details

Table 1 summarizes the input resolution, number of channels, and layer configuration at each stage of the backbone and head. Table 2 details the hyperparameters used for training, including optimizer, learning rate, batch size, and number of epochs.

## Appendix C. KeypointNet Architecture Details

Table 3 describes the resolution and the number of channels for each layer. Each row describes a stage *i* with layers, along with the input resolution 〈×〉 and the output number of channels . As shown in Table 4, the hyperparameters of the KeypointNet model used in all experiments include the optimizer AdamW, a learning rate of 1×, a batch size of 20, and 150 epochs.
